# smORFer: a modular algorithm to detect small ORFs in prokaryotes

**DOI:** 10.1093/nar/gkab477

**Published:** 2021-06-14

**Authors:** Alexander Bartholomäus, Baban Kolte, Ayten Mustafayeva, Ingrid Goebel, Stephan Fuchs, Dirk Benndorf, Susanne Engelmann, Zoya Ignatova

**Affiliations:** GFZ German Research Centre for Geosciences, Section Geomicrobiology, 14473 Potsdam, Germany; Inst. Biochemistry and Molecular Biology, Department of Chemistry, University of Hamburg, 20146 Hamburg, Germany; Inst. Biochemistry and Molecular Biology, Department of Chemistry, University of Hamburg, 20146 Hamburg, Germany; Helmholtz Center for Infection Research, Microbial Proteomics, 38124 Braunschweig, Germany; Inst. Microbiology, TU Braunschweig, Braunschweig, Germany; Inst. Biochemistry and Molecular Biology, Department of Chemistry, University of Hamburg, 20146 Hamburg, Germany; Robert Koch Institute, Berlin, Germany; Otto von Guericke University, Bioprocess Engineering, 39106 Magdeburg, Germany; Max Planck Institute for Dynamics of Complex Technical Systems, Bioprocess Engineering, 39106 Magdeburg, Germany; Helmholtz Center for Infection Research, Microbial Proteomics, 38124 Braunschweig, Germany; Inst. Microbiology, TU Braunschweig, Braunschweig, Germany; Inst. Biochemistry and Molecular Biology, Department of Chemistry, University of Hamburg, 20146 Hamburg, Germany

## Abstract

Emerging evidence places small proteins (≤50 amino acids) more centrally in physiological processes. Yet, their functional identification and the systematic genome annotation of their cognate small open-reading frames (smORFs) remains challenging both experimentally and computationally. Ribosome profiling or Ribo-Seq (that is a deep sequencing of ribosome-protected fragments) enables detecting of actively translated open-reading frames (ORFs) and empirical annotation of coding sequences (CDSs) using the in-register translation pattern that is characteristic for genuinely translating ribosomes. Multiple identifiers of ORFs that use the 3-nt periodicity in Ribo-Seq data sets have been successful in eukaryotic smORF annotation. They have difficulties evaluating prokaryotic genomes due to the unique architecture (e.g. polycistronic messages, overlapping ORFs, leaderless translation, non-canonical initiation etc.). Here, we present a new algorithm, smORFer, which performs with high accuracy in prokaryotic organisms in detecting putative smORFs. The unique feature of smORFer is that it uses an integrated approach and considers structural features of the genetic sequence along with in-frame translation and uses Fourier transform to convert these parameters into a measurable score to faithfully select smORFs. The algorithm is executed in a modular way, and dependent on the data available for a particular organism, different modules can be selected for smORF search.

## INTRODUCTION

Next-generation sequencing (NGS) technologies enable a rapid and easy detection of genomic information of new species. However, delineating protein-coding open reading frames (ORFs) in genomes after sequencing and *de novo* genome assembly remains still a challenge. After the pioneering effort of Fickett to unify concepts on how to define protein-coding sequences (1), further criteria have been added to increase the confidence in *de novo* identifications. These include intrinsic signals involved in gene specifications (e.g. start and stop codon, splice sites), conservation patterns in related genomes with weighted conservation depending on evolutionary distance and verification with known ORFs or protein sequences (2,3). Classically, these rules in the genome annotation protocols are performing well only on larger ORFs which span at least 100 codons (4,5), thus small ORFs (smORFs) shorter than 100 codons are systematically underrepresented and cannot be identified by common algorithms (6). Mounting evidence suggests crucial functions for smORFs in cellular and molecular processes in both eukaryotes (6–13) and prokaryotes (14–22). However, systematic identification of functional small proteins or microproteins (also called micropeptides) remains challenging both experimentally and computationally.

Recent developments of NGS technologies to probe the position of translating ribosomes with codon precision – ribosome profiling or Ribo-Seq (23), enable detecting actively translated ORFs by capturing ribosome-protected fragments (RPFs) and is used to empirically annotate coding sequences (CDSs). Several new previously unannotated ORFs, including smORFs, have been identified mostly in eukaryotes (8,24–26). Some studies oppose that RPFs alone are sufficient to classify a transcript as protein-coding or non-coding (27). Alternatively, Poly-Ribo-Seq which specifically sequences polyribosomes separated through sucrose gradients is suggested as more stringent approach in isolating translated ORFs (28). mRNAs translated by more than one ribosome (i.e. polyribosomes) are classically defined as genuinely translated mRNAs. However, studies in eukaryotes show that monosomes, initially considered as non-translating ribosomes, are in fact elongating ribosomes involved in translation of low-abundance transcripts or such with much slower initiation than elongation (29), or bear tissue-specific translation signature (30). Moreover, given that a ribosome protects on average 26–30 nt, this approach may miss a significant fraction of expressed transcripts and in particular very short smORFs (less than 10 amino acids) whose size might permit translation by a single ribosome, and thus, they migrate in the monosomal fraction. Ribo-Seq combined with an antibiotic treatment that specifically stalls ribosomes at translation initiation site (TIS-Ribo-Seq) selects for potential new initiation sites and allows detecting new ORFs in non-coding regions or overlapping ORFs which overlap with annotated ORFs and are undistinguishable in the Ribo-Seq data sets (8,19,22,25,31–34).

Complementing Ribo-seq with computational predictions revealed several hundred smORFs in eukaryotes (8,24,26,35,36). The crucial metrics they use, is the enrichment of RPFs in ORFs and the 3-nt periodicity characteristic for genuinely translating ribosomes. These approaches have difficulties evaluating prokaryotic genomes due to their unique architecture, including polycistronic messages, large fraction of overlapping ORFs, leaderless translation and lack of classical ribosome-binding site (i.e. with direct start of translation from the start codon (37,38)). The resolution of the prokaryotic Ribo-Seq data is lower than that in eukaryotes due to the intrinsic properties of the nucleases used in prokaryotic Ribo-Seq experiments (39), which often results in imperfect periodicity. Together, this makes a genome-wide identification of smORFs encoding functional small proteins in prokaryotes even more challenging.

Here, we present a new algorithm, smORFer, for identifying smORFs by integrating genomic information, structural features, Ribo-Seq and TIS-Ribo-Seq to faithfully select translated and initiated ORFs, respectively. The algorithm is executed in a modular fashion and various modules can be selected dependent on the data availability for each organism. smORFer is versatile and suitable for every organism, but shows high confidence of predictions for in particularly difficult-to-annotate smORFs in bacteria.

## MATERIALS AND METHODS

### Data sets used in the analysis

We generated two biological Ribo-Seq replicates for *Staphylococcus aureus* Newman and downloaded *Escherichia coli* MG1655 (Ribo-Seq, GSM3455899 and retapamulin-treated TIS-Ribo-Seq, GSM3455900 (19)) and *Bacillus subtilis* data (Ribo-Seq, GSM872395 and GSM872397, (40)) from the Gene Expression Omnibus (GEO) repository. The Ribo-Seq data for *S. aureus* Newman were uploaded in GEO under accession number GSE150601. Mass spectrometry data for *S. aureus* are from (41) and for *E. coli* from (42).

### Ribo-Seq of *S. aureus*

Cells grown in TSB medium (pancreatic digest of casein 17 g/l, enzymatic digest of soya bean 3 g/l, NaCl 5 g/l, K_2_HPO_4_ 2.5 g/l, glucose 2.5 g/l, pH 7.3) to OD_550_ = 1 were harvested by rapid centrifugation, resuspended in ice-cold 20 mM Tris lysis buffer pH 8.0, containing 10 mM MgCl_2_·6 H_2_O, 100 mM NH_4_Cl, 0.4% Triton-X-100, 4 U DNase, 0.4 μl Superase-In (Ambion), 1 mM chloramphenicol and disrupted by homogenisation (FastPrep-24 ™, MP Biomedicals) with 0.5 ml glass beads (diameter 0.1 mm). 100 A_260_ units of ribosome-bound mRNA fraction were subjected to nucleolytic digestion with 10 units/μl micrococcal nuclease (Thermofisher) in buffer with pH 9.2 (10 mM Tris pH 11 containing 50 mM NH_4_Cl, 10 mM MgCl_2_, 0.2% triton X-100, 100 μg/ml chloramphenicol and 20 mM CaCl_2_). The rRNA fragments were depleted using the *S. aureus* riboPOOL rRNA oligo set (siTOOLs, Germany) and the library preparation was performed as previously described (43).

### Data processing and mapping

Raw sequencing reads were trimmed using FASTX Toolkit (quality threshold: 20) and adapters were cut using cutadapt (minimal overlap of 1 nt). The following genome versions were used for mapping: *E. coli* U00096.3, *S. aureus* NC_009641.1 and *B. subtilis* NC_000964.3. Genomes and annotations were downloaded from NCBI (January 2020). In the first step of mapping, reads mapping to rRNAs were discarded. Thereafter, reads were uniquely mapped to the reference genomes using Bowtie (44), parameter settings: -l 16 -n 1 -e 50 -m 1 –strata –best. Non-uniquely mapped reads were discarded. The total number of mapped reads are summarized in Supplementary Table S1.

The peptide identification for *E. coli* was performed using the dataset PXD000498 (mascot_daemon_merge.mgf) (42) available at PRIDE (45). To identify a MASCOT (version 2.6) (46) search against the smORF candidates (taking only longest smORF for candidates sharing the same stop codon) and all protein coding genes (4,243 sequences) and the respective decoy database was carried out with search parameters as previously published (42).

### smORFer workflow

**Figure 1.**
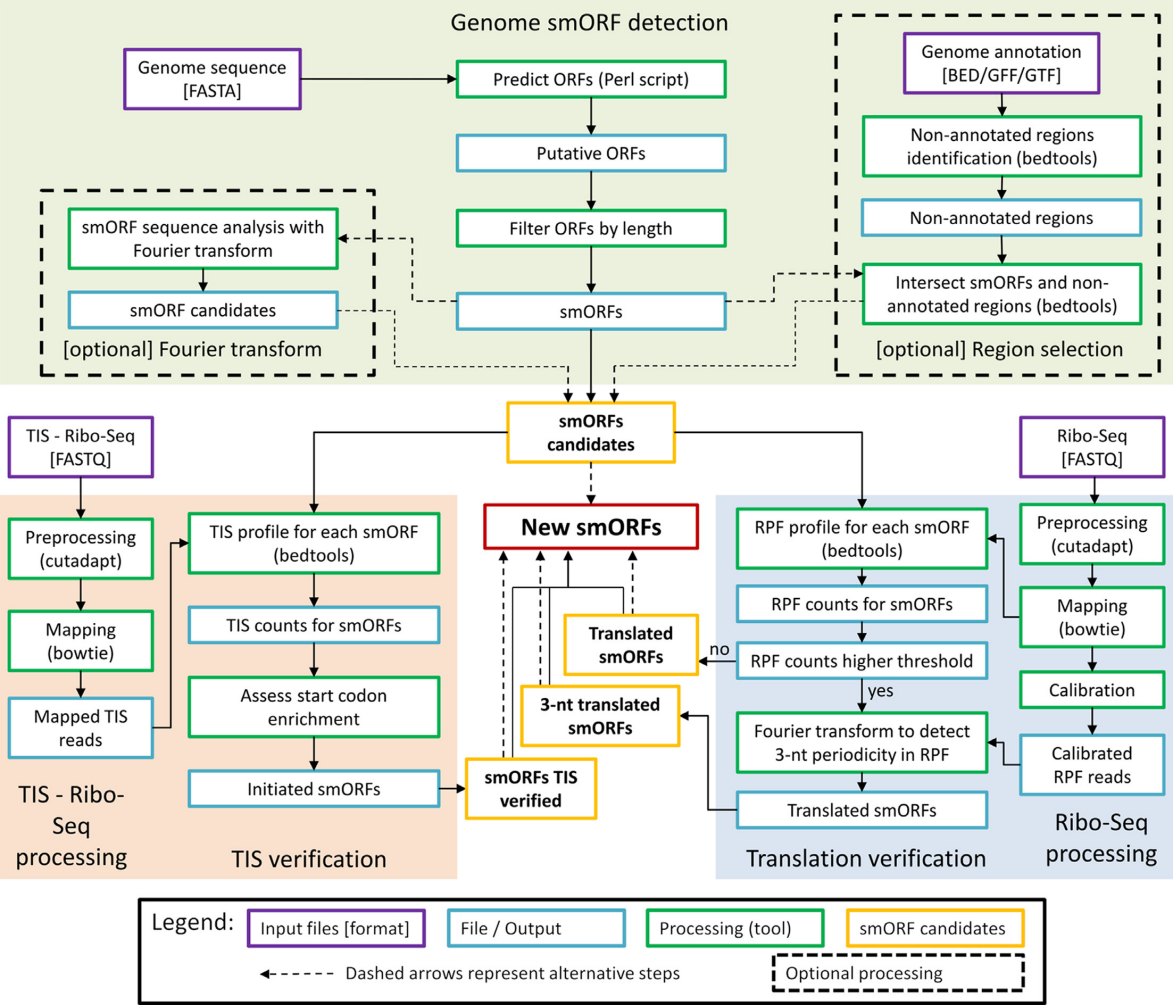
General scheme of smORFer algorithm with its three modules that evaluate genomic information (module A, green), translation and 3-nt periodicity in the RPFs from Ribo-Seq data (module B, blue), and TIS from TIS-Ribo-Seq (module C, orange).

The workflow of smORFer, which is executed in a modular way, is summarized in Figure 1. Several simple counting and filtering steps are performed using BEDTools (47), e.g. ORFs in non-annotated regions where filter by intersectBed and counting read was done using coverageBed. The first part of Module A is required to define the boundaries of all putative ORFs. The selection is further refined by the structural properties that are intrinsic to protein-coding sequences. Modules B and C add further confidence to the detected smORF candidates and can be executed either independently or together; the latter increases the detection of true positive novel smORFs.

#### Genome-based ORF detection (Module A)

A list of putative ORFs was generated using modified Perl script (48); it generates putative ORFs with in-frame start and stop codon. We used four start codons, ATG, GTG, TTG, CTG, which are the most common in prokaryotes (49), and the three uniform stop codons, TGA, TAG, TAA. smORFer separated smORFs based on their location, e.g. in the non-annotated and annotated regions, and also contains a strand-specific filter for selecting the region.

To detect whether a putative smORF potentially encodes peptides or proteins, i.e. exhibits 3-nt *sequence periodicity* of the CDS, and hence, would be potentially translated, we used Fourier transform (FT; implemented as R’s base fft function) of the GC content of each single gene, i.e. for each single ORF this is a vector of 0’s and 1’s. The signal is first normalized to the ORF length as the signal intensity depends on the ORF length. In this 3-nt periodic pattern the 1.5-nt period is always present along with the 3-nt period regardless of the length of the putative ORF. Thereafter, we build the fraction of normalized signal at the period of 3 nt and divide it by the arithmetic mean of the signal between both 3 nt and 1.5 nt periods.

#### Detection of translated ORFs from Ribo-Seq data including read processing (Module B)

Ribo-Seq data are first mapped and smORFs with a minimum of five RPFs are selected and assigned as ‘*translated’*. A coverage of ≥5 RPF counts is on average above the counting error for short ORFs in Ribo-Seq data sets (23,50) and we suggest it to be used as an arbitrarily cutoff when biological replicates are not available. Otherwise, the reliable minimum read counts per gene should be determined individually for each Ribo-Seq using variability analysis of the counting statistics of two independent biological replicates that also assesses the influence of counting noise (23,43).

The calibration procedure assigns for each RPF the codon at the ribosomal A or P site, allowing for tracking the codon-wise periodic pace of ribosomes along ORFs. To position a read at the ribosomal A or P site, the reads are first binned by length and the offset is determined for each read length bin individually as described ((51); all scripts are available here: https://github.com/AlexanderBartholomaeus/MiMB_ribosome_profiling). For prokaryotic Ribo-Seq data sets, a calibration using 3′ ends, i.e. to the termination codons, is recommended since the nucleases used to generate RPFs in bacteria cleave in a sequence selective manner with somewhat less variations at the 3′ ends (52). The read length distributions vary between data sets likely because of different experimental protocols (53) and at least four to five highest read length bins should be considered. Here, we considered for *E. coli* and *B. subtilis* read length bins of 27–30 nt and for *S. aureus* 24–28 nt bins with an offset for the A site of 11 nt for 24–28 nt and 12 nt for 29–30 nt. Alternatively, other algorithms that extract A or P site from the RPF reads can be used. Similarly to our approach, Plastid (54) and RiboProfling (55) compute the P site by stratifying the reads in bins according to their length and treating each bin independently yield variable offsets across bins. riboWaltz (56), a two-step R algorithm, computes the P site with a high accuracy using a coherent single offset.

Calibration requires good read coverage, hence smORFs with a coverage of 100 RPFs per kilobase of ORF length (RPK) were further subjected to FT analysis to determine the 3-nt or codon periodicity of the calibrated RPF profile. Usually, a coverage of 100 RPK (i.e. 1 read per 10 nt) results in a good FT analysis. smORFs with a 3-nt periodicity in the RPF coverage are classified as ‘*3-nt translated’*. Next, the 3-nt or codon periodicity of the calibrated RPF profile is subjected to FT and a score is extracted from the mean of the signal between the periods of 3 nt and 1.5 nt. The threshold (FT > 2) is determined from the cumulative distributions of FT values for 2,315 protein-coding ORFs with ≥100 RPK. smORFs with a FT value higher of 2 are classified then as ‘*3nt-translated’*. smORFs with low RPF coverage, for which a 3-nt periodic signal in the RPF profiles could not be determined, are sorted as *‘translated’*. Note, that *‘translated’* smORFs should be also kept as they could be true hits, but their relatively low translation levels, with only few RPFs, preclude calibration and FT analysis.

#### Detection of TIS (Module C)

Ribo-Seq is performed in the presence of translation initiation inhibitor; here, for *E. coli* retapamulin was used (19). TIS-Ribo-Seq was processed in the same way as Ribo-Seq. The middle nucleotide of each RPF is extracted and used in further analysis; in the case of even read length, the 3′ nucleotide of the first half of an RPF is taken (51). It should be noted that reads from TIS-Ribo-Seq cannot be calibrated, because of the skewed coverage at initiation and the lack of coverage at termination; the latter prevents calibration at both start and stop codons (51). Moreover, a manual assignment of the offset is not possible, because retapamulin binds to the peptidyl-transferase center in both presence and absence of initiator fMet-tRNA (19,57,58), thus blurring the P-site assignment over at least two codon positions. For each smORF, the middle-nucleotide TIS counts over the three nucleotides of the start codon and one codon upstream and downstream of the start are summed up and ORFs with more than 5 RPFs are classified as having true TIS.

### Operating system and R versions, scripts and examples

We used Ubuntu 18.04 LTS as the operating system. For data analysis and visualization, we used R (3.5.0) including packages seqinr (3.6-1) and Biostrings (2.50.2) which are available on all operating system. Scripts, example calls and files (except BAM files because of their large size) for smORFer using *E. coli* data sets are available at https://github.com/AlexanderBartholomaeus/smORFer.

## RESULTS AND DISCUSSION

### Design of the smORFer: a modular algorithm to detect smORFs

The availability of various sequencing data (DNA-Seq, Ribo-Seq, TIS-Ribo-Seq) for different organisms may largely vary, hence we sought to develop an algorithm—smORFer—with a modular design which uses various data sets to detect putative smORFs. smORFer combines three modules which utilize different inputs and can be used independently or in combination to increase the confidence in smORFs annotation (Figure 1). The three inputs are: (i) the genomic nucleotide sequence for module A ‘*Genome-based smORF detection’*, (ii) Ribo-Seq data for module B ‘*Detection of translated ORFs’* and (iii) TIS-Ribo-Seq for module C ‘*Detection of TIS*’ (Figure 1).

#### A. Genome-based ORF detection

This module uses genomic data as an input to first predict putative ORFs in a length-independent manner. In all three organisms tested, we detected a large number of putative smORFs with a length between 3 and 50 codons (including the stop codon). We restricted the maximal length cutoff to ≤50 codons; the length of 50 codons has been defined for the category of small or micropeptides (22,59). The algorithm, however, can perform calls for ORFs at any length. A single amino acid ORF, although theoretically possible to be produced from a start-stop-ORF (19), does not fulfil the criteria for a peptide and was not considered. We used 3 codons (i.e. including start and stop codons) as it will encode the shortest peptide, i.e. a dipeptide. We used four start codons, ATG, GTG, TTG, CTG, which are the most common in prokaryotes (49), and the three uniform stop codons, TGA, TAG, TAA.

**Figure 2.**
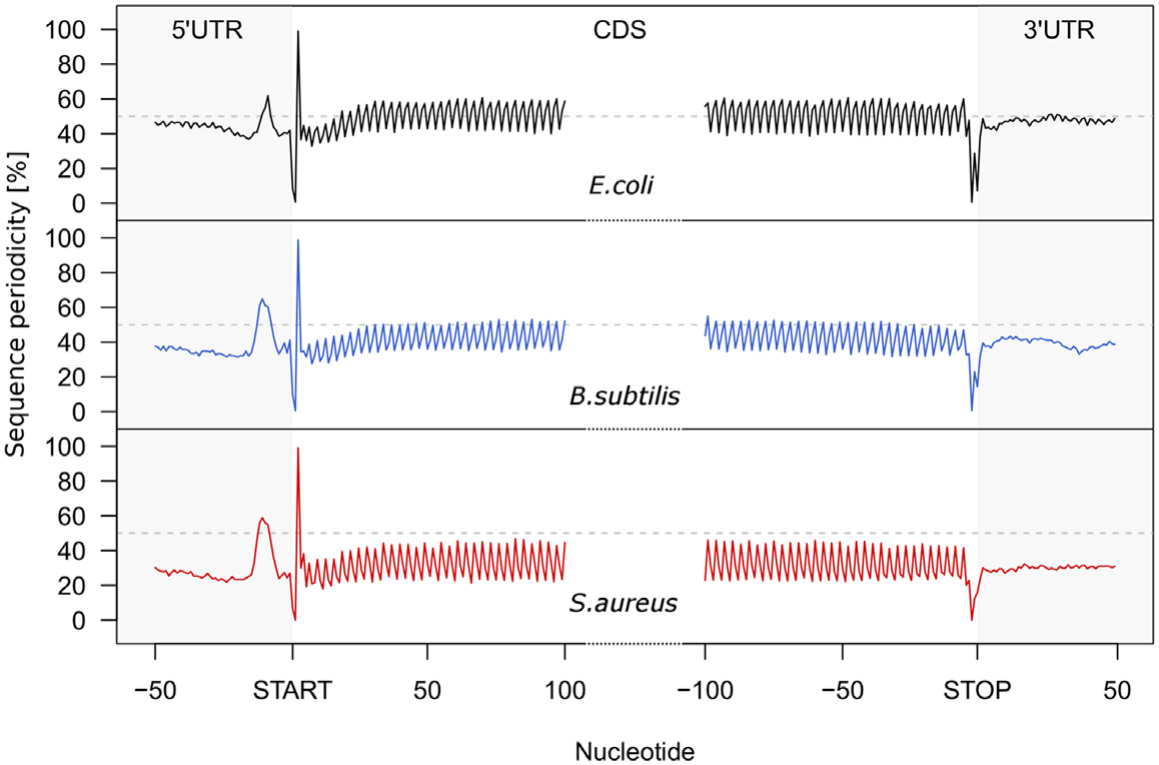
Metagene analysis of the *genomic sequence periodicity* across the 5′UTRs, CDSs and 3′UTRs of all protein-coding transcripts in *E. coli* (black), *B. subtilis* (blue) and *S. aureus* (red). ORFs are aligned at the start or stop codon, respectively. Note that the GC content differs among organisms and is 51% for *E. coli*, 44% for *B. subtilis* and 33% for *S. aureus*. Only non-overlapping protein-coding ORFs are considered. The horizonal dashed line denotes the average structure of a hypothetical genome with 50% GC content.

**Figure 3.**
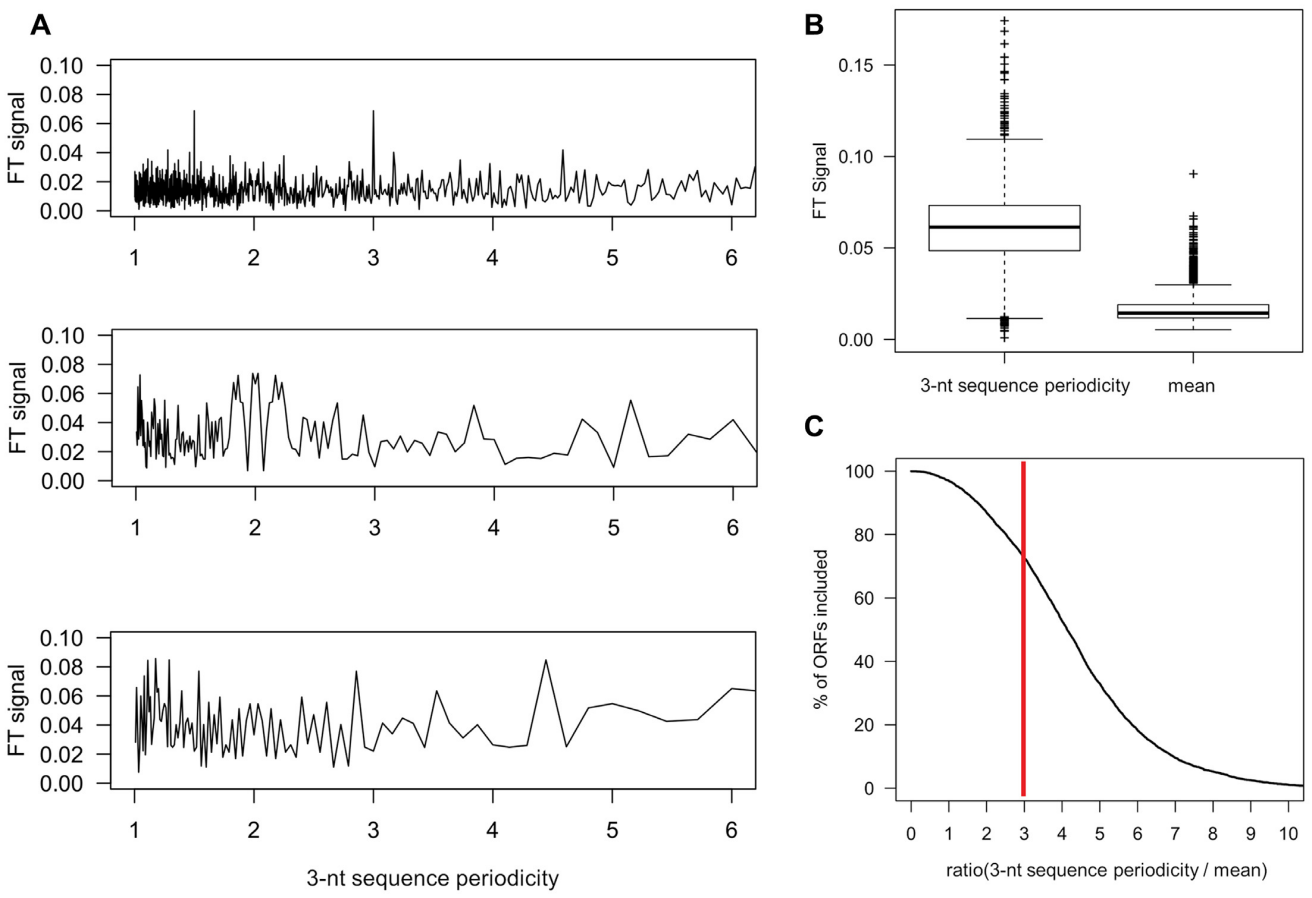
Fourier transform (FT) of the genomic *3-nt sequence periodicity* in ORFs. (**A**) Averaged *3-nt sequence periodicity* of protein-coding ORFs (upper panel), intergenic region (middle panel) and non-protein coding gene (e.g. 5S rRNA, lower panel). (**B**) The FT signal normalized by the ORF length at *3-nt sequence periodicity* (left) and by the arithmetic mean of the signal between periodicity at 1.5 and 3 (right) for protein-coding ORFs. (**C**) Cumulative distributions of FT values (from B) for protein-coding ORFs. Vertical red line, cutoff of 3.

Analysis of the genomes from the three organisms revealed a well-defined *3-nt sequence periodicity* within the genomic DNA sequences of the CDSs (Figure 2), which is a characteristic feature of protein-coding CDSs (60). Next, we included a step to assess the discrete genomic *3-nt sequence periodicity* of each putative ORF using Fourier transform (Figure 3A, B, Supplementary Figure S1). To decrease the number of false-positives, we used a relatively restrictive cutoff (FT > 3, Figure 3C) which detects appr. 70% of known ORFs. For comparison, a cutoff of 2 would detect 85% of the known ORFs.

#### B. Detection of translated ORFs from Ribo-Seq data

**Figure 4.**
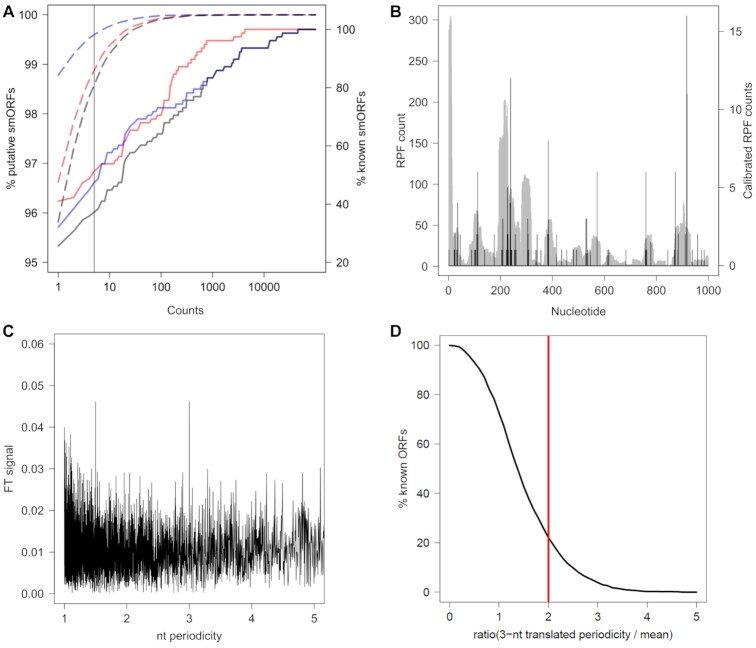
Detected smORFs for Ribo-Seq and TIS-Ribos-Seq data for *E. coli* and FT analysis of calibrated RPF counts. (**A**) Fraction of putative smORFs (dashed lines, left axis) and known smORFs (solid lines, right axis) (22) that are detected as *translated* and with genuine TIS with smORFer. Note the linear dependence of known smORFs that is caused by their different expression levels, while the putative smORF show a non-linear dependence. Red, *translated* (i.e. RPF counts); blue, with genuine TIS (i.e. RPF counts at TIS); black, both *translated* and with TIS counts. Vertical line denotes the cutoff ≥5 RPFs. (**B**) RPF reads plotted in full length (grey, left axis) for the first 1000 nt of the *RNase I* transcript compared to the calibrated RPF counts (black, right axis). (**C**) 3-nt periodicity FT signal of the calibrated RPFs for *RNase I* transcript. (**D**) Cumulative distributions of FT values for protein-coding ORFs. Vertical red line, cutoff of 2.

This module assesses the translation of each ORF from a Ribo-Seq data set (Figure 1). First, to filter out ORFs with a translation level below the threshold of sporadic expression, smORFer selects ORFs with a minimal coverage (≥5 RPFs) and categorizes them as *translated*. This threshold, inferred from earlier data sets (23,50) provides a good balance of true false-positives and false-negatives as revealed by the comparison with experimentally verified smORFs (Figure 4A). Genuinely translated ORFs exhibit a 3-nt periodicity in their RPF coverage, hence at a second stage, ORFs undergo a 3-nt periodicity analysis which is assessed again using FT (Figure 4B, C). smORFs over the threshold (FT > 2, Figure 4D) are categorized as *3-nt translated*. In the FT analysis of the calibrated RPFs we used a restrictive cutoff (FT > 2, Figure 4D) which detects 512 of the known protein-coding ORFs in *E. coli*. Usually the 3-nt pattern is well detectable in smORFs with a good coverage, yet we do not discard smORFs with no discernible periodic RPF coverage (*translated* category) as they could be still expressed but translated at low level.

#### C. Detection of TIS

This module uses as input TIS-Ribo-Seq data. To block the ribosomal transition from initiation into elongation and to detect bona fide initiating ribosomes in prokaryotes, several antibiotics have been used so far, e.g. retapamulin (19), Onc112 (22), tetracycline (33). Thereby, retapamulin shows the sharpest peak at initiation. Considering the middle nucleotide of each TIS-Seq read and summing up TIS counts at the start codon including one codon upstream and downstream (Supplementary Figure S2), smORFer selects smORFs with ≥5 RPFs at TIS (the same cutoff as for *translated* above) and categorizes them as *translated* with TIS signal (Figure 1, Table 1).

**Table 1.** Comparison of the results for smORFs in three different organisms. For *E. coli* MG1655 Ribo-Seq and TIS-Ribo-Seq are available, whereas for *B. subtilis* and *S. aureus* only Ribo-Seq is available. Modules desigantion as in Figure 1

Module	Description of the filtering step	*E. coli*	*S. aureus*	*B. subtilis*
Genome-based smORF detection (module A)	smORFs length ≥9 to ≤150 nt	415 133	305 549	405 321
	smORFs in non-annotated regions	234 399	179 679	238 446
	*3-nt sequence* periodicity detected by Fourier transform	10 689	9608	13 619
Detection of translated ORFs (module B)	*translated* smORFs with ≥5 RPF	3079	6595	2105
	*3-nt translated* smORFs with 3-nt RPF periodicity detected by Fourier transform	175	555	168
Detection of TIS (module C)	with ≥5 RPFs at start codon	424	n.a.	n.a.
Cross comparison	*translated* smORFs and TIS-Ribo-Seq signal	160	n.a.	n.a.
	*3-nt translated* smORFs with 3-nt RPF periodicity and TIS-Ribo-Seq signal	16	n.a.	n.a.

### Performance of smORFer for *de novo* identification of smORFs

Here, we employed the smORFer in predicting smORFs in three different organisms, *E. coli*, *B. subtilis* and *S. aureus*. For all three organisms Ribo-Seq data are available, and TIS-Ribo-Seq only for *E. coli*. In all three microorganisms tested, based on the genomic sequence and using the first search criterion, we detected a large number of putative smORFs with a length between 3 and 50 codons (>300 000, Table 1). Selection by this simple feature (48) revealed a large portion of overlapping smORFs, i.e. smORFs with different start codons but terminated by the same stop codon. Four different start codons, ATG, GTG, TTG and CTG, which are the four most used in bacteria, were used as selection criterion. Thereby, in *E. coli* their usage differs by several orders of magnitude, e.g. the usage is ATG 81.8%, GTG 13.8%, TTG 4.34% and CTG 0.024% (49). This start codon usage has been deduced from annotated (large) ORFs, but since smORFs may follow non-canonical codons and rules of initiation (37,38), we kept all four codons with equal weight in the search. This initial step is required to set the boundaries of all possible putative ORFs.

Compared to the 5′ and 3′ UTRs, the coding sequences (CDS) of all three organisms exhibit a well-defined *3-nt sequence periodicity* which is independent of the GC content of the organism (Figure 2). Even *S. aureus* genome with the lowest GC content (33% GC content) shares the same 3*-nt periodic sequence* pattern. Thus, we reasoned that smORFs, if protein or peptide coding, would share the same *3-nt sequence periodicity* like annotated long CDSs encoding large proteins. To extract the *3-nt sequence periodicity* of the genomic sequence, we subjected all smORFs to FT analysis which converts this characteristic pattern into a score (Figure 3 and Supplementary Figure S1) and used it as a further filtering criterion in module A (Table 1). Because of quite short smORF length and lower signal-to-noise ratios (61), we used a stringent FT score to select smORFs potentially encoding peptides (FT > 3, Figure 3C). Even with this stringent criterion, although it significantly reduced the number of potential candidates, the number of smORFs remained relatively large (Table 1).

Again, the majority of the detected smORFs in this step remained the overlapping ones, i.e. with distinct start codons but terminated by the same stop codon. Within these, the distribution of smORFs initiated with ATG, GTG, TTG and CTG was for *E. coli* 900, 625, 815 and 739, for *B. subtilis* 702, 422, 609 and 372, and for *S. aureus* 2356, 1212, 2227 and 800, respectively. Notably, the distribution among the start codons in the putative smORFs is relatively balanced between these four start codons unlike their skewed distribution in initiating long annotated ORFs (49). At this stage, in order to not miss non-canonically initiated smORFs, we do not apply further selection criteria.

Next, using Ribo-Seq data we analyzed the translation status of the smORFs with *3-nt sequence periodic pattern* (module B, Figure 1). In total, 3079, 6595 and 2105 smORFs for *E. coli*, *S. aureus* and *B. subtilis*, respectively, were selected with RPFs over the threshold (named *translated* candidates, Table 1). Overall, the identified smORFs were translated at very low level, exhibiting only few RPFs. Next, we applied a more stringent criterion for selecting genuinely translated ORFs and assessed the 3-nt periodicity of their RPF profile which is a characteristic feature of a genuine translation. For this, the RPFs were precisely positioned within ORFs, or calibrated by aligning their 3′ ends to the stop codons (52)—a key step in obtaining a codon resolution and extract 3-nt periodicity of the RPF profile. The calibrated RPFs to the ribosomal A site were then subjected to FT analysis which converts this 3-nt characteristic pattern of the RPF coverage into a score. smORFs with FT ≥2 were defined as *3-nt translated* candidates (Table 1). In this step, 175, 555 and 168 non-annotated smORFs were discovered in *E. coli*, *S. aureus* and *B. subtilis*, respectively (Table 1).

For *E. coli*, a TIS-Ribo-Seq data set using retapamulin to stall imitating ribosomes was available (19), which we used for further verification of both *translated* and *3-nt translated* categories in module B (Table 1). Inspection of the TIS coverage in the annotated protein-coding ORFs showed that retapamulin crisply stalls over the start codon including one codon upstream and downstream of it, with a maximum coverage centered over the start codon (Supplementary Figure S2). From the 3079 *translated* smORFs, 160 possessed a TIS signal and from the 175 *3-nt translated* – 16 (Table 1). The marked reduction of the number of potential candidates from these selected in the *translated* category emphasizes the importance of using various data sets to enhance stringency and confidence in smORFs identification and select genuinely translated candidates from translational noise.

**Figure 5.**
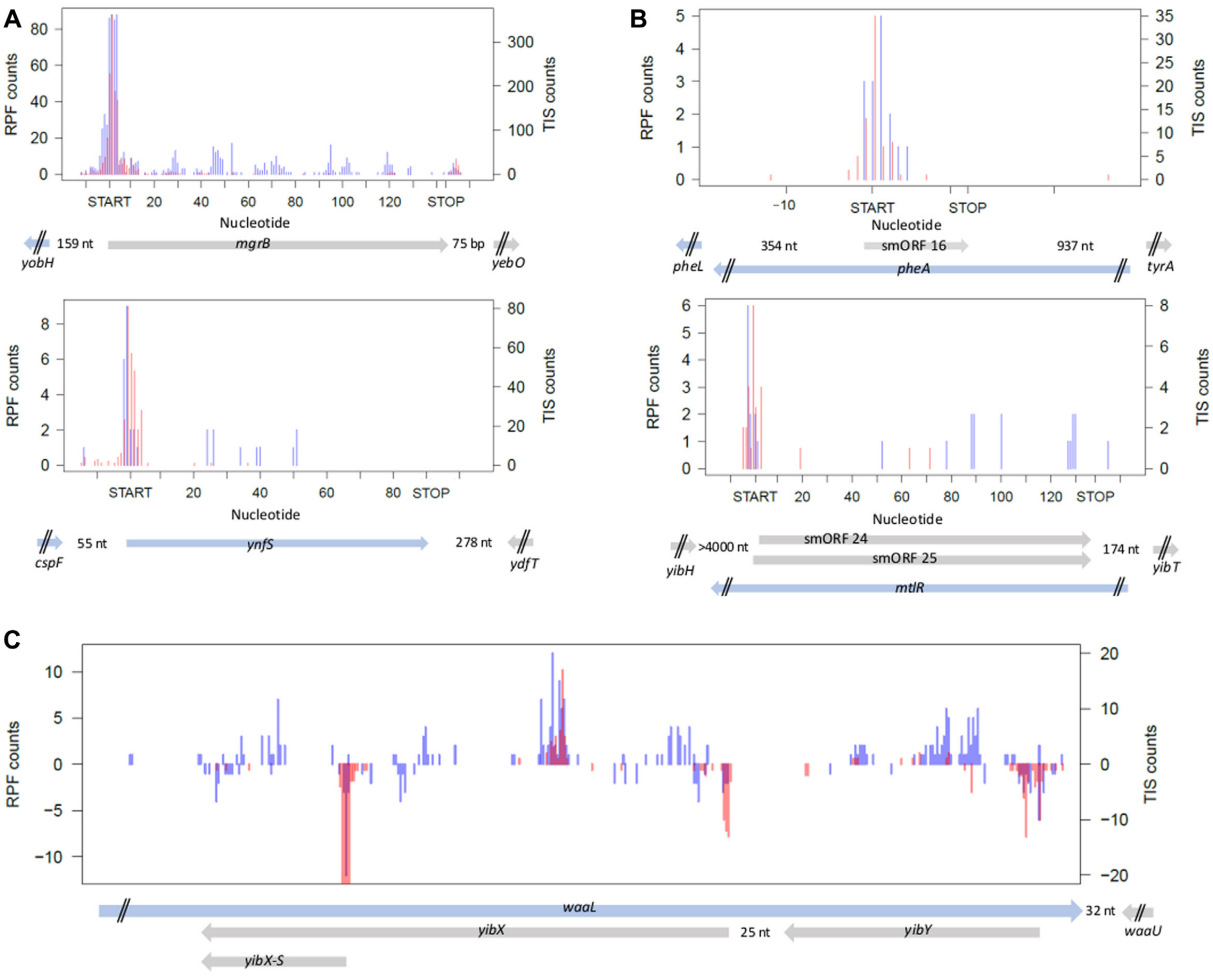
Examples of known and newly detected smORFs with smORFer in *E. coli*. (**A**) Examples of known and already experimentally verified smORFs (22) detected also with smORFer. (**B**) Examples of newly identified smORFs from each category, *translated* (upper panel) and *3nt-translated* (lower panel). Lower panel: smORFer predicted two smORFs that differ only by their adjacent start codons. Since TIS-Seq counts are spread ± one codon around the start codon (Supplementary Figure S2), there is no clear-cut indication for a preferred start. smORF 25 has two consecutive start codons (both TTG) and is by one start codon longer than smORF 24, otherwise both smORFs are identical. (**C**) Complex example of smORFs overlapping with known ORFs illustrating strand-specificity of RPF and TIS-Seq counts, and precise identification of smORF translational start site. All 3 smORFs, including also the short *yibX-S* version of *yibX*, are detected by smORFer and experimentally verified in (22). Counts displayed as positive values of the y-axes represent counts of ORFs located on the forward DNA strand, and negatively displayed counts of ORFs on the reverse strand. (A–C) Blue, RPF counts from the Ribo-Seq (left axis); red, counts from the TIS-Seq (right axis). ORFs architecture is shown at the bottom: blue arrow, ORFs located on the forward strand; gray, ORFs located on the reverse strand.; nt, denotes the distance to the next ORF; two black dashes, designate truncated, not-completely displayed adjacent ORFs.

In *E. coli*, using all three modules the algorithm successfully detected the experimentally verified smORFs, including also some recently identified smORFs with manually assessed TIS (19,22) (Figure 5A and Supplementary Figure S3). Notably, the number of detected known smORFs by both, RPF and TIS signal, increased linearly with the RPF counts of smORFs (Figure 4A), suggesting an expression-dependent effect. smORFer detected new smORFs (Figure 5B), many of which were overlapping and counted as independent in the modules A and B (Table 1). The true power of TIS-Ribo-Seq is in the precise positioning of the likely true start codon and thus, selecting true translated smORFs from overlapping frames (Figure 5C). Since TIS and RPF data are strand specific, we can clearly distinguish signals from each DNA strand, thus, precisely assigning smORFs (*yibX*, *yibX-S*, *yibH*) on the opposite strand to the *waaL* ORF (Figure 5C). smORFer is also able to unambiguously assign overlapping ORFs on the same strand (Figure 5C), given that the TIS signals are separated by minimum 3 nt (Supplementary Figure S2). The algorithm may miss some cases of completely overlapping ORFs, initiated through adjacent start codons (<3nt, Figure 5B), although long stretches of overlap among ORFs are fairly rare (Supplementary Figure S4). It is worth mentioning, that retapamulin is so far the only initiation inhibitor for Gram-negative bacteria, that exhibits such precise inhibition at start codons (19) and allows for exact detection of TIS. Other antibiotics show much broader coverage across initiation sites and are not always precisely centered at the initiation codon (22,33). To decrease the false-positive hits, in particular for very short smORFs, we recommend executing restrictive call with a coverage over the start codon and expand it by maximally one codon at each side.

In all three categories, i.e. the ORFs detected by the genome-based search, *translated* and *3-nt translated* smORFs, which were verified in the *E. coli* TIS-Ribo-Seq data set (Table 1), we analyzed the distribution of the start codons. Among the 424 smORFs with TIS signal, the distribution of the smORFs initiated with ATG, GTG, TTG and CTG was—229, 65, 92 and 38, respectively. While for the 160 *translated* smORFs the distribution among the initiation codons was similar—79, 24, 43 and 14, respectively, for the *3-nt translated* 16 candidates this changed to—5, 1, 7 and 3, respectively. This clearly distinct usage of start codons in smORFs, even within the most stringent—the *3-nt translated* group, suggests that smORFs exhibit a different bias of start codon usage than long protein-coding ORFs (49). Based on this distribution, it is conceivable to include a start-codon selection step in module A; however, TIS-Ribo-Seq data are available for only one organism and ideally this distribution, if uniform among prokaryotes as expected, should be experimentally verified for other bacteria.

For *E. coli—*the most studied organism—we sought to consider other datasets to further cross validate the predictions of smORFer. We used the most extensive mass spectrometry dataset available for the *E. coli* proteome (42). Using a Mascot score >100 and FDR <0.01 (46), from the total of 4245 protein-coding genes in *E. coli*, we detected 1890 proteins (∼44.5%). Applying the same criteria to the detected smORFs (1 unique peptide, 7 duplicates, best matching score 44), we detected only one candidate; 6 further candidates were selected with a score <100 (single peptides) (Supplementary Figure S5). The Ribo-Seq, TIS-Ribo-Seq and mass spectrometry show a good overlap in detecting known protein-coding genes (Supplementary Figure S6A), yet the depth of Ribo-Seq and even TIS-Ribo-Seq are much higher than mass spectrometry, that is likely the reason to limit the detection of smORFs. Most of the smORFs are expressed at much lower levels compared to long protein-coding ORFs (Supplementary Figure S6B). Furthermore, the trypsin-generated fragments are also non-unique, e.g. more than 2500 of the 3079 *E. coli* smORFs do not exhibit any unique peptide with ≥6 amino acids (Supplementary Figure S6C).

We also considered recently published mass spectrometry data for *S. aureus* (41) and detected smORFs with *SALT* & *Pepper* pipeline (https://gitlab.com/s.fuchs/pepper) (41). The pipeline uses a minimum of one unique peptide larger than 6 amino acids detected in at least two biological replicates and with a minimum score of 40 for unmodified and modified peptides, a minimum delta score of 6 for unmodified peptides and 17 for modified peptides, and a fixed false discovery rate (FDR) of 0.0001 for peptides and 0.01 for proteins (41). The *SALT* & *Pepper* pipeline comprises genomic prediction of smORFs and mass spectrometry verification and detected in total 176 unique small proteins with a length of up to 100 amino acids. Thereby, 144 of them passed our criteria for genuinely translated, i.e. ≥5 PRF as in the category for *3-nt translated* smORFer candidates; 32 did not have any RPFs and were likely false-positives detected in *SALT* & *Pepper*. Among the 144 candidates, 17 were with a length ≤ 50 amino acids which is a selection criterion in smORFer.

Despite this fairly low overlap between the smORFs in the category *3 nt-translated* (Table 1) and detected by mass spectrometry, it is of the same order of magnitude as observed for smORFs translated in Ribo-Seq data and detected by mass spectrometry for human cell lines (8). Besides the higher depth of the sequencing-based data than the mass spectrometry (62), several other reasons may contribute to the larger numbers of smORFs identified by deep-sequencing approaches than by mass spectrometry (Supplementary Figure S6 and S7): (i) low expression level of smORFs, (ii) lack of unique peptides to be detected and uniquely assigned to smORFs, (iii) too short peptidase-generated peptides to be detected by mass spectrometry, and (iv) conditional expression of smORFs under particular stress despite their constant translation in the translation noise. It should be also noted that mass spectrometry pipelines detect mainly soluble (small) proteins leaving out a large fraction of membrane or membrane-anchored proteins; the latter represent a significant fraction among small proteins (5,38).

### Comparison of the performance of smORFer to other tools

We compared smORFer to RibORF (63,64) and GETORF (65). Similarly to smORFer, RibORF utilizes a multistep procedure, including Ribo-Seq data for detecting translated ORFs, similar minimal smORF length of 9 nt, thus, it is the most appropriate algorithm for comparison. GETORF is part of the EMBOSS suite and finds ORFs based on the nucleotide sequence and was chosen to compare the performance in detecting putative ORFs in the genomic module of smORFer (Figure 1). We performed three different comparisons: (i) general detection of putative smORFs from genomic sequences, (ii) detection of long ORFs which are usually well captured in various algorithms, and (iii) detection of smORFs in non-annotated regions. Notably, with the first criterion (i) smORFer and RibORF detected identical number of putative ORFs from the genomic sequences which was higher than these generated by GETORF (Table 2). Since both smORFer and RibORF detect ORFs with multiple starts but sharing the same stop codon, the number of detected ORFs is reduced when only stop codons were counted, i.e. considering one ORF per stop codon (Table 2).

**Table 2.** Comparison of the performance of smORFer with RibORF and GETORF in genome-based search for putative smORFs. The minimal and maximal length of smORFs was set at 9 nt and 150 nt, respectively

Algorithm	*E. coli*	*S. aureus*	*B. subtilis*
GETORF	180 278	202 597	200 992
RibORF	415 133	305 549	405 321
smORFer	415 133	305 549	405 321
unique stop codons RibORF/smORFer	176 427	148 408	179 274

The second comparison (ii) evaluates the results of RibORF and smORFer in detecting long ORFs, i.e. >1000 nt, in the *E. coli* genome. Using genomic sequence, both RibORF and smORFer predicted a large number of putative ORFs, which however was much higher than the annotated ORFs in *E. coli*, as multiple start codons were considered which share the same stop codon (Table 3). Counting the ORFs by unique stop codon, 99.6% of the known annotated ORFs were detected by both algorithms. Including further criteria to select for translated ORFs, RibORF detected 235 translated ORFs (1.2% of all known ORFs >1000 nt) compared to 740 (45% of all known ORFs >1000 nt) detected by smORFer. In part, this is due to the utilization of TIS-Ribo-Seq data, emphasizing the importance of using such data sets to precisely map initiation sites. It should be noted that RibORF, which does not use TIS-Ribo-Seq, runs much slower than our algorithm (2 days versus 6 h).

**Table 3.** Comparison of the performance of smORFer and RibORF in identifying long ORFs (>1,000 nt) in *E. coli*

RibORF results		Overlap with known ORFs	Overlap with total known ORFs, %^c^
ORFs in genome	38 284 (1,811)^a^	1576^b^	99.6
3-nt periodicity Ribo-Seq	26 850 (1,185)	1122	70.9
predicted ORFs	235 (235)	19	1.2
smORFer results		Overlap known ORFs	Overlap with total known ORFs, %
ORFs in genome	38 284 (1,811)^a^	1576^b^	99.6
*translated* ORFs ≥5 RPFs	32 949 (1,483)	1381	87.3
ORFs ≥5 RPFs at start	912 (746)	717	45.3
*translated* ORFs and ≥5 RPFs at start	906 (740)	711	44.9

^a^In brackets the ORFs counted by unique stop codons.

^b^Some ORFs are not found by the genome search because they have non-canonical start or stop codons.

^c^In total, there are 1582 annotated ORFs with length >1000 nt in *E. coli*.

Third (iii), we compared RibORF and smORFer by scanning only the non-annotated regions. RibORF detected 42, 463 and 1178 smORFs for *E. coli*, *B. subtilis* and *S. aureus*, respectively (Table 4). The number of validated smORF candidates by smORFer were higher 3079, 2105 and 6595 for *E. coli*, *B. subtilis* and *S. aureus*. Notably, for *B. subtilis* and *S. aureus* many smORFs predicted by RibORF overlapped with the set of *translated* candidates detected with smORFer (Table 4). The number of *3-nt translated* smORFs was much lower and showed no overlap to the RibORF final candidates. While RibORF identified precisely larger ORFs (Table 3), smORFer outperformed it in detecting smORFs. This behavior is likely a result of the underlying assumption of RibORF which similarly to other algorithms identifies new ORFs using the same assumptions used for long ORFs (66), namely 3 nt periodicity as a diagnostic of a *bona fide* translation and protein conservation (63,64). Many experimentally verified bacterial peptides encoded by smORFs exhibit different composition bias than the proteome encoded by long ORFs (67). In contrast, our results reveal characteristic features of smORFs that differ for long ORFs emphasizing on the importance of adjusting the selection criteria to the features of smORFs.

**Table 4.** Comparison of the performance of smORFer and RibORF in detecting smORFs (≥9 and ≤150 nt) within the non-annotated regions

RibORF		Overlap	smORFer	
*E. coli*				
smORFs	234 399	234 399	234 399	smORFs
3-nt periodicity	546	-	-	-
predicted ORFs	42	41	3079	*translated* ≥5 RPFs
		0	175	*3-nt translated*
		2	160	*translated* ORFs and ≥5 TIS counts at start
		0	16	*3-nt translated* ORFs and ≥5 TIS counts at start
*B. subtilis*				
smORFs	238 446	238 446	238 446	smORFs
3-nt periodicity	1176	-	-	-
predicted ORFs	463	458	2105	*translated* ≥5 RPFs
		0	168	*3-nt translated*
*S. aureus*				
smORFs	179 679	179 679	179 679	smORFs
3-nt periodicity	1821	-	-	-
predicted ORFs	1178	1168	6595	*translated* ≥5 RPFs
		0	555	*3-nt translated* ORFs

## CONCLUSION

Comprehensively designed for annotating *de novo* smORFs using various data sets, smORFer presents remarkable advantages. It has a high efficiency in predicting smORFs with high probability to be expressed. The modularizable structure of smORFer offers advantages in verifying the smORFs calling dependent on the available data sets for each organism. The first part of module A, the genome-based ORF detection, is imperative as a starting point, since it sets the genomic boundaries of smORFs. The 3*-nt sequence periodicity* detection (FT, module A) decreases the search space and we recommend using it when no further deep-sequencing data is available for the particular organism. For organisms, for which Ribo-Seq and/or TIS-Seq data are available, we recommend after the genomic search in module A to directly process with modules B and/or C. Both modules B and C can be applied independently dependent on the available deep-sequencing data sets: the higher the number of the data sets and the modules run in smORFer, the higher the accuracy of the smORF prediction. TIS-Ribo-Seq is particularly powerful in unambiguously assigning overlapping smORFs.

Deep-sequencing-based approaches offer higher depth than mass spectrometry. However, to decrease the number of false-positives, several approaches should be combined (i.e. Ribo-Seq combined with TIS-Ribo-Seq to select for genuine initiation, and/or with Term-Seq for determining faithful termination (68)). Sequencing approaches delineating initiation and termination are in particular useful in genomes with overlapping reading-frames architecture as the prokaryotic genomes. smORFer is also suitable for eukaryotes; we recommend running module A on the transcriptome since eukaryotic genomes can reveal extremely large number of smORFs requiring a large computation power.

Many smORFs might be expressed only under stress conditions. Hence, the next challenge is to surgically dissect their expression with Ribo-Seq and TIS-Ribo-Seq collected under various stress conditions. This will allow conditionally translated smORFs to be disambiguated from the pool of smORFs with no RPFs under permissive conditions, i.e. categorized as untranslated. When paired to smORFer such data sets, expression events, even conditional expression events, will be mapped more comprehensively.

Computationally, smORFer enables full analysis in a standardized way requiring little computational resources. The workflow in each module is easy to use and simple to modify to achieve high precision in smORFs calling.

## DATA AVAILABILITY

Raw sequencing data including the RPM tables have been deposited within Gene Expression Omnibus (GEO) under accession number GSE150601.

All scripts used for the calibration of RPFs are available at https://github.com/AlexanderBartholomaeus/MiMB_ribosome_profiling. Scripts, example calls and files for smORFer using *E. coli* data sets are available at https://github.com/AlexanderBartholomaeus/smORFer.

## Supplementary Material

gkab477_Supplemental_File
