# Supplementary material for: smORFer: a modular algorithm to detect small ORFs in prokaryotes
Source: Nucleic Acids Res. 2021 Jun 14;49(15):e89. doi: 10.1093/nar/gkab477 (PMC8421149; doi:10.1093/nar/gkab477)
Supplement: gkab477_Supplemental_File [file gkab477_supplemental_file.pdf]

SUPPLEMENTARY INFORMATION

Table S1. Mapping outcome for different data sets.

Organism	Total number of sequencing reads	Uniquely mapped reads
E. coli strain MG1655 (1)		
Ribo-Seq	65,014,082	11,054,240
TIS-Ribo-Seq	36,693,777	6,534,911
S. aureus strain Newman (this work)		
R#1 Ribo-Seq	26,221,544	3,366,465
R#2 Ribo-Seq	20,114,374	2,334,161
B. subtilis strain 168 (2)		
R#1 Ribo-Seq	18,230,224	6,807,640
R#2 Ribo-Seq	35,407,892	12,531,457

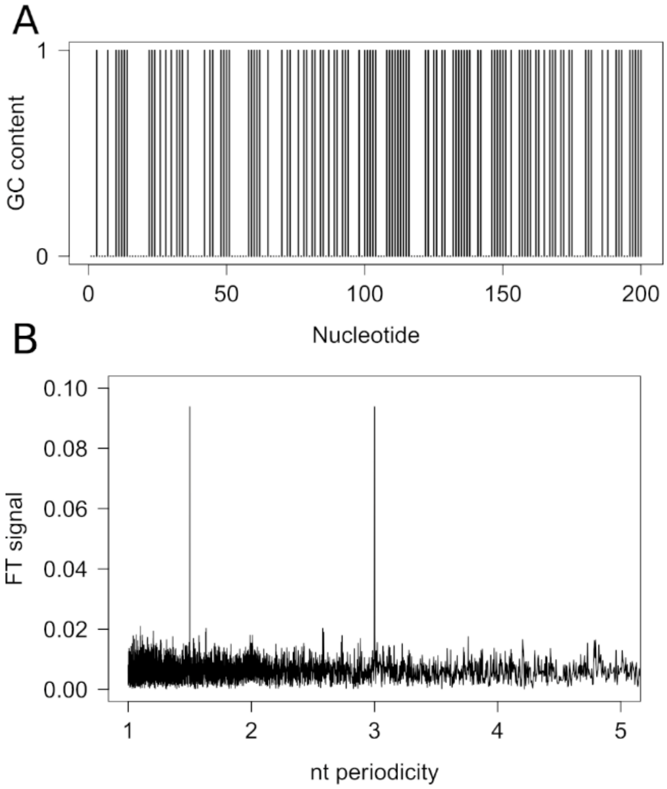

Figure S1. *3-nt sequence periodicity* of a single gene example. (A) Untransformed and (B) FT-transformed sequence periodicity of *RNase I* gene. In panel A the first 200 nt are shown. The *structural 3-nt periodicity* is only detectable following FT.

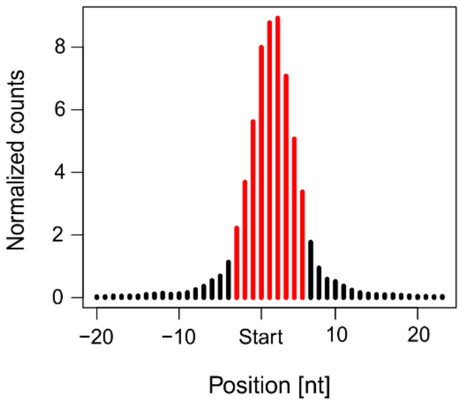

Figure S2. TIS-Ribo-Seq counts are enriched at the start. Counts for known ORFs, represented by their middle nucleotide, are normalized and each ORF is equally weighted. TIS counts show a clear enrichment at the start codon including one adjacent codon up- and downstream of it. Start denotes the first nucleotide of the start codon. Red, one codon up- and downstream of the start codon.

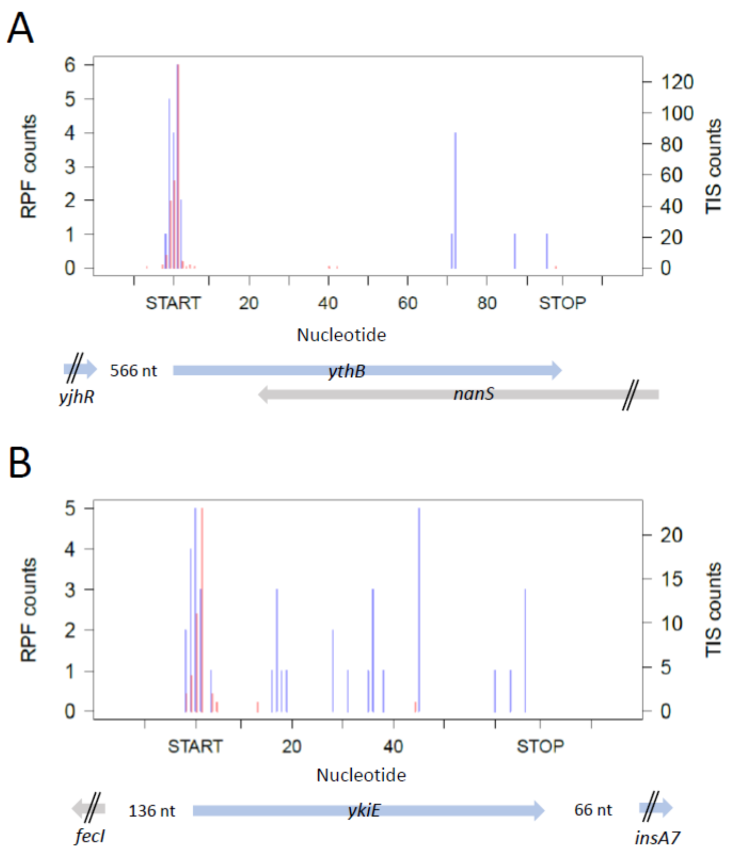

Figure S3. Examples of two smORFs detected by smORFer and also identified using manual assessment of TISs (3). The red counts represent the counts from TIS-Ribo-Seq and blue from the Ribo-Seq. Blue, PRF counts from the Ribo-Seq (left axis); red, counts from the TIS-Seq (right axis). ORFs architecture is shown at the bottom: blue arrow, ORFs located on the forward strand; gray, ORFs located on the reverse strand; nt, denotes the distance to the next ORF; two black dashes, designate truncated, not-completely displayed adjacent ORFs.

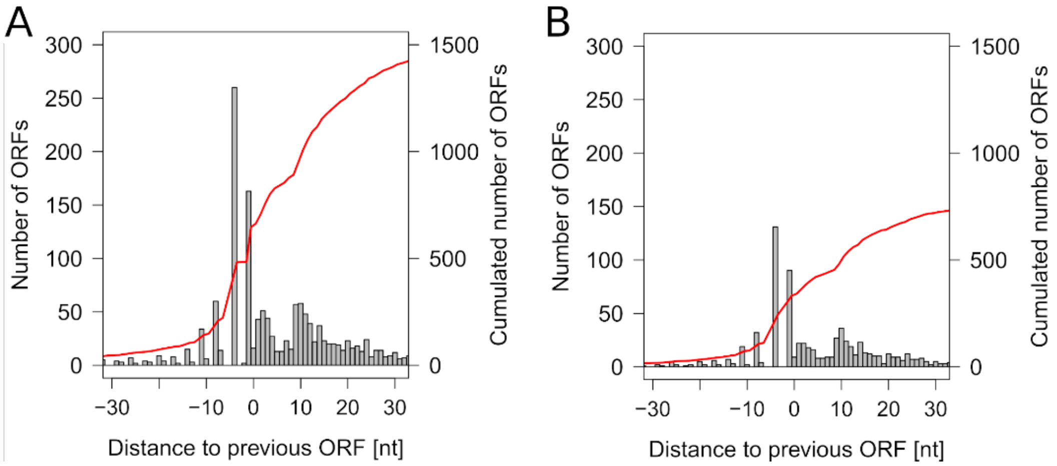

Figure S4. Overlap of known *E. coli* ORFs based on distance to previous ORFs. (A) Overlap when considering ORFs on both strands. Cumulatively, 146 overlaps of ≥ 10 nt are found up to -10 nt (x axis). (B) Overlap when considering only ORFs on the same strand. There are 76 overlaps of ≥ 10 nt. Red line, cumulative plot of ORFs number (right y-axis). Note that only a minor fraction of ORFs are overlapping in *E. coli* when considering overlap on the same strand.

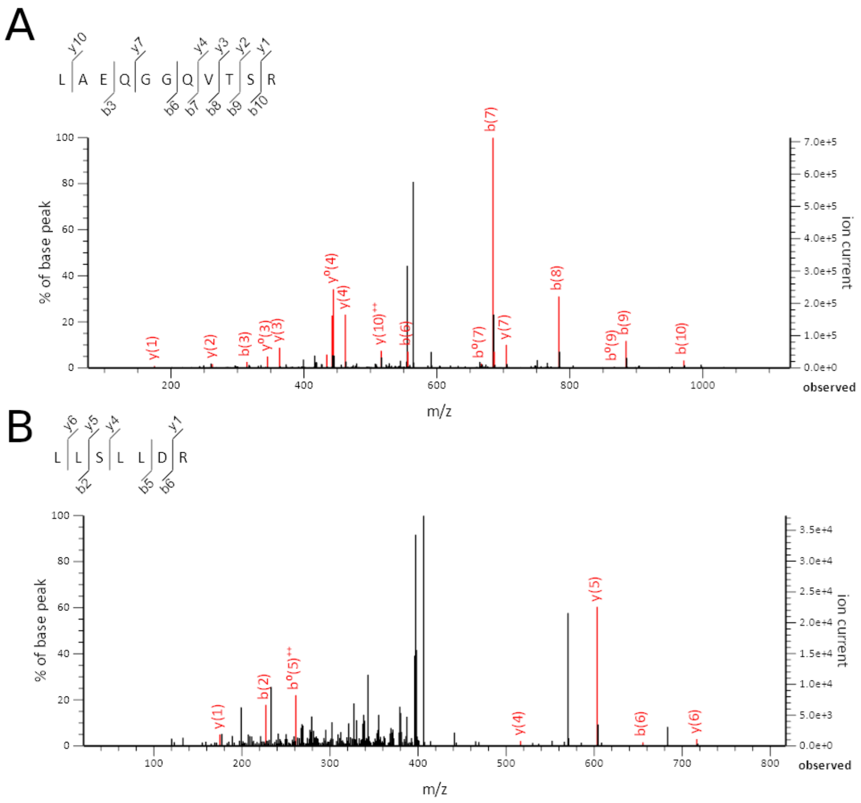

Figure S5. Mass spectrometry (MS) spectrum of a newly identifies smORF. (A) Spectrum for a smORF that is clearly verified by MS with fragments matching for all abundant fragments. The few non-matching fragments can be explained by neutral losses. (B) Spectrum of a smORF non-verified by MS, with only few matching fragments but several non-annotated high-abundance fragments.

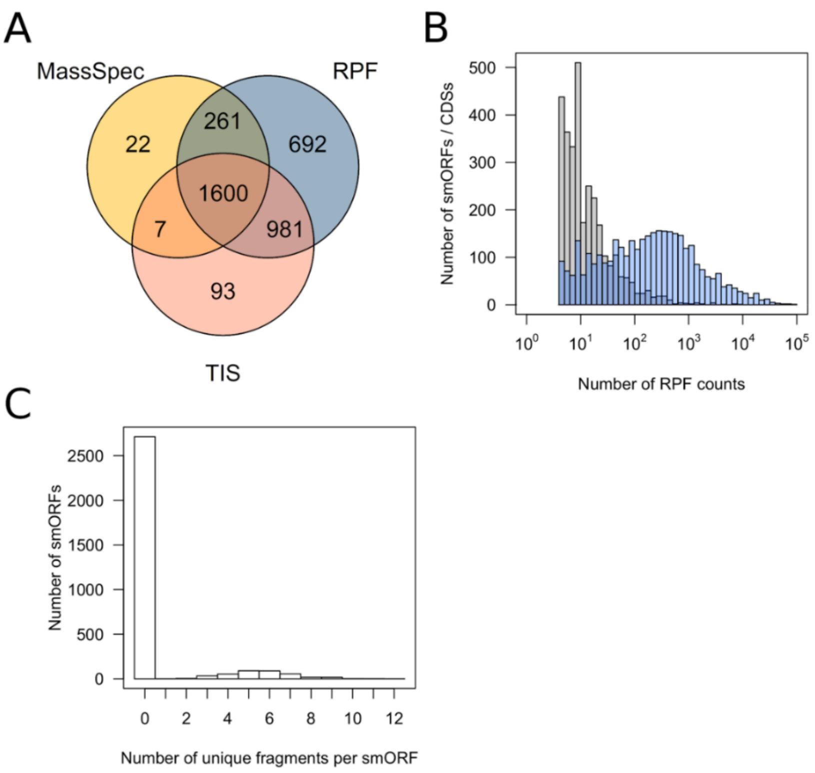

Figure S6. Comparison of the proteomics detection and smORFer for *E. coli*. (A) Overlap of the number of detected protein-coding genes by mass spectrometry (MassSpec), Ribo-Seq (RPF) and TIS-Ribo-Seq (TIS). (B) Expression level of smORFs (grey) and protein-coding genes (blue) as detected from the RPF counts in Ribo-Seq. (C) Putative unique peptides (> 6 amino acids) generated by tryptic digestion of smORFs compared to all smORFs with ≥5 RPFs.

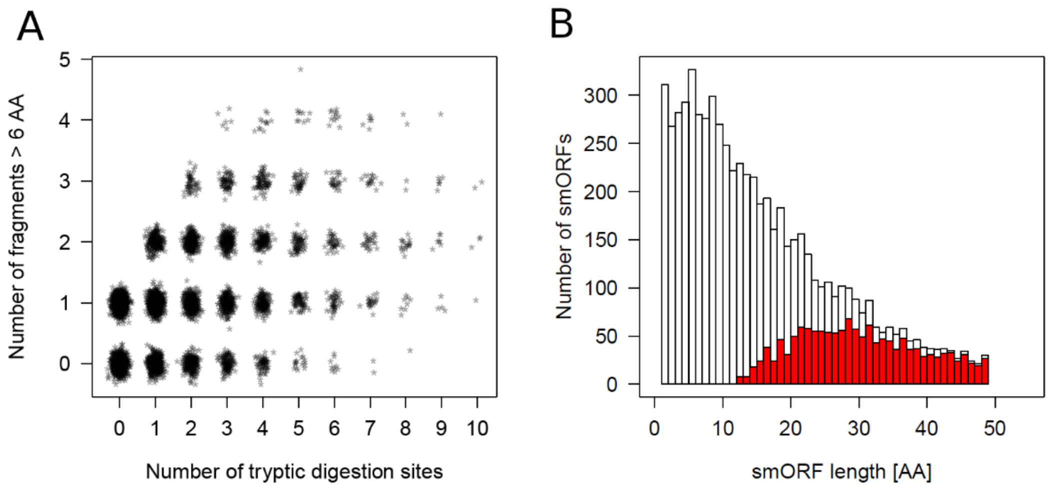

Figure S7. Putative trypsin cleavage sites within the *translated* 6,595 smORFs candidates in *S. aureus* (Table 1). (A) The number of fragments generated by trypsin cleavage from each smORF. (B) Distribution of smORFs length. The red designated smORFs are the subset of all 6,595 candidates that fulfil the *SALT&Pepper* algorithm threshold of at least two peptides with a length of 6 amino acids (4).

SUPPLEMENTARY REFERENCES

1. Meydan, S., Marks, J., Klepacki, D., Sharma, V., Baranov, P.V., Firth, A.E., Margus, T., Kefi, A., Vazquez-Laslop, N. and Mankin, A.S. (2019) Retapamulin-Assisted Ribosome Profiling Reveals the Alternative Bacterial Proteome. *Mol Cell*, **74**, 481-493 e486.
2. Li, G.W., Oh, E. and Weissman, J.S. (2012) The anti-Shine-Dalgarno sequence drives translational pausing and codon choice in bacteria. *Nature*, **484**, 538-541.
3. Weaver, J., Mohammad, F., Buskirk, A.R. and Storz, G. (2019) Identifying Small Proteins by Ribosome Profiling with Stalled Initiation Complexes. *mBio*, **10**.
4. Fuchs, S., Kucklick, M., Lehmann, E., Beckann, A., Wilkens, M., Kolte, B., Mustafayeva, A., Ludwig, T., Diwo, M., Wissing, J. *et al.* (2020) A proteogenomics workflow to uncover the world of small proteins in Staphylococcus aureus. *bioRxiv*.
